# Prevalence and risk of occurrence of visible birth defects in mining areas in South Kivu: A hospital-based cross-sectional study

**DOI:** 10.1371/journal.pone.0309004

**Published:** 2024-10-07

**Authors:** Fabrice Gulimwentuga Cikomola, Alliance Wani Bisimwa, Arsene Daniel Nyalundja, Ernest J. Barthélemy, Bijoux Safi Matabaro, Franck Masumbuko Mukamba, Kinja Nyamugabo, Désiré Munyali Alumeti, Théophile Kabesha, Marc Miserez, Dieudonné Mushengezi Sengeyi

**Affiliations:** 1 Department of Surgery, Hôpital Provincial Général de Référence de Bukavu (HPGRB), Bukavu, South Kivu, Democratic Republic of Congo; 2 Faculty of Medicine, Catholic University of Bukavu, Bukavu, South Kivu, Democratic Republic of Congo; 3 Center for Tropical Diseases and Global Health, Catholic University of Bukavu, Bukavu, South Kivu, Democratic Republic of Congo; 4 Division of Neurosurgery, Global Neurosurgery Laboratory, SUNY Downstate Health Sciences University, Brooklyn, NY, United States of America; 5 School of Public Health, SUNY Downstate Health Sciences University, Brooklyn, NY, United States of America; 6 Faculty of Medicine, Université Officielle de Bukavu, Bukavu, South Kivu, Democratic Republic of Congo; 7 Department of Surgery, KU Leuven-University Hospital Leuven, Leuven, Belgium; 8 Faculty of Medicine, Department of Gynaecology and Obstetrics, University of Kinshasa, Kinshasa, Democratic Republic of the Congo; National Research Centre, EGYPT

## Abstract

**Background:**

Possible contamination related to mining activities might contribute with other risk factors in increasing the burden of birth defects (BDs) in many developing countries including the Democratic Republic of Congo. The subsequent prevalence is frequently underestimated. Implementation of focused public health interventions is hindered by the paucity of comprehensive data. We assessed the potential impact of mining on the prevalence and occurrence of visible BDs in neonates in South Kivu (SK).

**Methods:**

A hospital-based cross-sectional study was conducted among 65,474 newborns registered in 7 hospitals in SK from 2016-2021. Hospitals were categorized based on mining activities in their respective catchment areas. Living in a mining zone was the exposure, whereas the outcome was visible BDs. Prevalence was estimated per 100,000 live births, and risk of occurrence with odds ratio (OR) and their 95% confidence interval.

**Results:**

261 neonates with visible BDs were recorded accounting for a prevalence of 399 cases per 100,000 live births. The prevalence ranges between 217 and 1365 cases per 100,000 live births. An increased risk was found in mining zones(OR=2.07; 95%CI=1.59-2.68), Mubumbano(OR=1.72, 95%CI=1.22-2.43), and Mwenga(OR=3.89, 95%CI=2.73-5.54), whereas a reduced risk was reported in non-mining zones(OR=0.48, 95%CI=0.37-0.62) in Katana (OR=0.49, 95%CI=0.33-0.73). Musculoskeletal(28.74%) and central nervous systems(19.92%) were the most common BDs. A significant difference in prevalence for BDs involving the face, GI system and abdominal wall, musculoskeletal, central nervous and genitourinary systems between mining and non-mining zones was found(*p<0*.*001*).

**Conclusion:**

There is an excessive risk for visible BDs in areas with hazardous mining activities in SK region.​​ More complex studies are needed to define the possible causal relationship. Moreover, findings generated herein should be corroborated by other research design, periodically monitored by public health authorities, and used to inform initiatives promoting enhanced environmental health, access to pediatric surgical care, and public health campaigns aimed at decreasing risk of BDs.

## Introduction

Birth defects (BDs) are structural or functional abnormalities of prenatal origin that arise during the developmental process [1]. They are one of the main causes of death and disability among neonates and children under five [2]. Each year, BDs affect an estimated 8 million newborns and are responsible for 10% of total deaths and disability adjusted-life years (DALYs) worldwide in children under five [3,4]. Nearly three million of cases are major BDs, with 90% occurring in developing countries, and are responsible for 495,000 deaths [5]. Although BDs contribute significantly to the burden of disease and generate a heavy socio-economic burden due to diagnosis and management challenges, they are often neglected and left far behind infectious diseases in the global public health discourse [6]. Moreover, most developing countries struggle to provide optimal antenatal and neonatal care. Rural and remote communities face barriers in accessing timely, affordable, quality and safe BDs care [7,8].

South Kivu (SK), in the eastern part of the Democratic Republic of Congo (DRC), hosts one of the most important mining deposits of the country after the Sahaba mining deposit in Katanga, which is a part of the African Copper belt. The province is abundant in minerals such as gold, coltan, cassiterite, wolfram, tourmaline and more, most of which are still extracted on an artisanal basis. In addition, one of its mining areas, Kamituga, hosts some of the country’s most important artisanal gold mines [9]. Although minerals represent one of the region’s main sources of revenue, they are smuggled out of the province in large-scale operations, often involving armed actors and rebel groups. Their extraction results in political conflicts and environmental issues such as deforestation, siltation of waters, soil degradation, dust pollution, poaching of wild animals and water pollution by products including acids, copper, lead, arsenic, cyanide and/or mercury, which are used for their purification or separation from other metals [10,11]. Miners and residents of urban areas near mining sites, especially children, are found to have very high concentrations of heavy metals in their urine and blood, as well as evidence of exposure-related oxidative DNA damage [12].

While the etiology of BDs is multifactorial including genetic, socioeconomic, and environmental factors, the reproductive and developmental toxicity of metals is increasingly recognized [13]. Specific metals such as mercury are posited as potentially the main cause of BDs [14]. Moreover, these conditions significantly contribute to the burden of disease in children with a prevalence between 453 and 584 cases per 100,000 live births [15,16]. BDs stand as one the top 5 leading causes of neonatal mortality and morbidity in Congolese, accounting for 8% all-cause mortality and admissions respectively [17,18]. However, evidence on BDs landscape in SK inhabitants remains sparse. Congolese clinicians and experts believe that existing data portray an inaccurate landscape because of underdiagnosis, inadequate information management systems, and limited access to quality prenatal and neonatal care. Sustainable improvement of outcomes for infants and children in SK with BDs requires accurate measurement of the burden of the disease, and the unmet need for comprehensive medico-surgical care of these children.

This study aims to describe the prevalence of BDs and assess the link between their occurrence and mining in SK, in the eastern region of the DRC. Specifically, the present manuscript investigates the distribution of BDs in mining and non-mining areas, and then compares the overall distribution to the ecological data from the Global Burden of Disease (GBD).

## Methods

### Study design, period, and setting

This cross-sectional study collected retrospective data on childbirth from the medical records of women who delivered in SK between January 2016 and August 2021. SK, a post-conflict province of 8,424,706 habitants in the eastern part of the DRC, is administratively divided into seven territories and 34 health zones (HZ). Each HZ includes a general reference hospital that provides an essential and comprehensive package of health services. The province is located within the Great Rift Valley; west of Burundi, Rwanda and Tanzania; east of Maniema province (DRC), south of North Kivu (DRC); and North Tanganyika province (DRC).

The lack of reliable data on maternal and infant health indicators in the region raises a concern, as it hampers accurate assessment and effective policymaking. The reported fertility rate stands at 7.6%, it’s noteworthy that 93.5% of pregnant women have access to at least one antenatal visit [19]. However, the coverage for antenatal care at least four times appears relatively low at 35.30%, and only 8.9% of deliveries occur at home [19]. The reported national neonatal mortality rate of 26 per 1,000 live births [20], with over 70% occurring in the early neonatal period [21]. The leading causes of neonatal mortality include prematurity, birth asphyxia, birth trauma, and neonatal sepsis. Additionally, consanguinity has a significant impact on birth outcomes, and has been reported in BDS, with half of cases involving first-degree relatives [22].

Study sites were selected on a convenience basis, and included four HZ in non-mining areas including Kadutu, Walungu, Katana and Panzi and 3 HZ in mining areas including Mwana, Mumbumbano, and Mwenga. These mining sites were selected based on their known intense mining activity.

### Study population

A total of 65,474 living neonates, including 14,424 in mining zones and 51,050 in non-mining zones, were registered at birth from January 2016 to August 2021 in the General Referral Hospitals of the aforementioned sites. Visible BDs were determined based on the clinical diagnosis made by the attending physician or midwife following delivery. All records with incomplete information were excluded, as well as stillbirth and immediate death after birth that did not occur at the study site.

#### Definitions of study variables

*Visible BDs*. Major BDs encompass structural changes that carry profound implications including surgical, medical, social and/or cosmetics, for the affected neonate and require comprehensive care [23–25]. They play a major in mortality, morbidity, and disability among neonates. Conditions such as spina bifida, hydrocephalus, anencephaly, heart defects, laparoschisis, sirenomelia (mermaid syndrome), orofacial clefts and more epitomize the category of major BDs. In contrast, minor BDs denote structural changes that typically entail minimal health concerns, exerting limited social or cosmetic impact on the neonate [23–25]. Minor types include, but not limited to, a single palmar crease, undescended testis, and umbilical hernia. BDs underwent further categorization based on the affected system: musculoskeletal, central nervous, digestive and abdominal wall, face, and urogenital systems. Neonates who were born with two or more BDs were designated as having a polymalformative syndrome, denoted as polymalformation. Major BDs occurring alongside a minor one was classified as major, whereas a combination of minor BDs was categorized as minor.

*Exposure*. Mining zone was considered as any HZ having one or more mining operation sites. These mining operation sites are required to be situated within the administrative boundaries of the HZ. Non-mining zones were deliberately chosen at a distance from mining zones to mitigate the odds of including individuals with a medium or suboptimal level of exposure, given the proximity between mining and non-mining zones. The primary mineral extracted in these mining areas is gold. However, Mwenga also has significant coltan and cassiterite mining, while Mubumbano is notable for its wolframite extraction.

### Source of data

Data on deliveries were collected from the medical registration records of the gynecology and obstetrics wards. Data collection period spanned from January 9, 2022 to March 31, 2022. The data collection tool was reviewed and validated by an expert team consisting of an obstetrician, a pediatrician, surgeons, and a researcher with formal training in data management. Two general practitioners trained in data collection tools and procedures were involved in data collection. We only included data on visible BDs.

### Statistical analysis

The prevalence of BDs was estimated as the proportion of living neonates, the numerator, among the total number of all neonates, the denominator, admitted during the study period. Then, the proportion was multiplied by 100,000 to determine the prevalence of BDs per 100,000 living births and was rounded to two decimal places.

Statistical analysis including univariate (frequency and percentage for categorical variables), and bivariate (chi-square test, t test of student, Wilcoxon rank-sum test, binary logistic regression), and multivariable analyses (Friedman Test and Friedman post-hoc Wilcoxon test with Bonferroni correction). The correction in multivariable analysis was used to assess the difference in prevalence among study sites. Only comparisons among groups with a significant alpha value (*p<0*.*05*) after the correction were considered a true difference. Association between the occurrence of BDs and mining was assessed through a regression model. The results from the model were summarized by odd ratios (OR) with related 95% confidence intervals (CI). A significant risk was considered for 95%CI not containing the null value (OR=1). All statistical analyses were conducted in SPSS 29 (IBM, WA USA).

### Ethical clearance

The ethical approval was obtained from the local Institutional Review Board (IRB) of the Catholic University of Bukavu (UCB) (**Ref. N° UCB/CIES/NC/020/2021**). Authorization letters were obtained from different hospital direction bureaus. The requirement for the informed consent was waived due the retrospective design of the study. The data collection began afterwards. Collected data was kept confidential and only research team members were granted access to it. Also, any information facilitating the individual identification of the newborns and their mother was expunged, and the authors were precluded from accessing it.

## Results

### Overall prevalence

**Fig 1:**
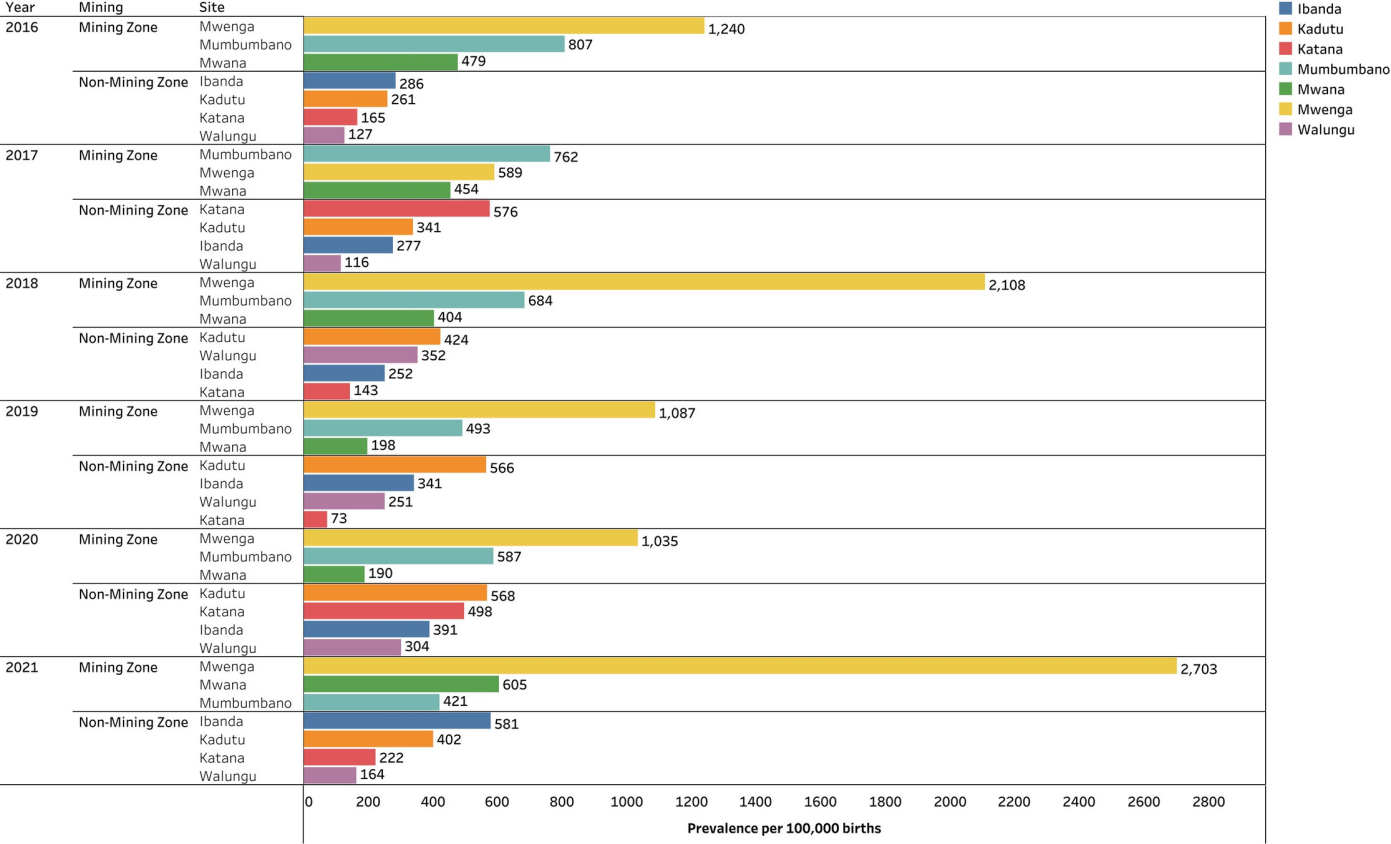
Bar chart of the prevalence of BDs per 100,000 births by study site from 2016 to 2021.

**Fig 2:**
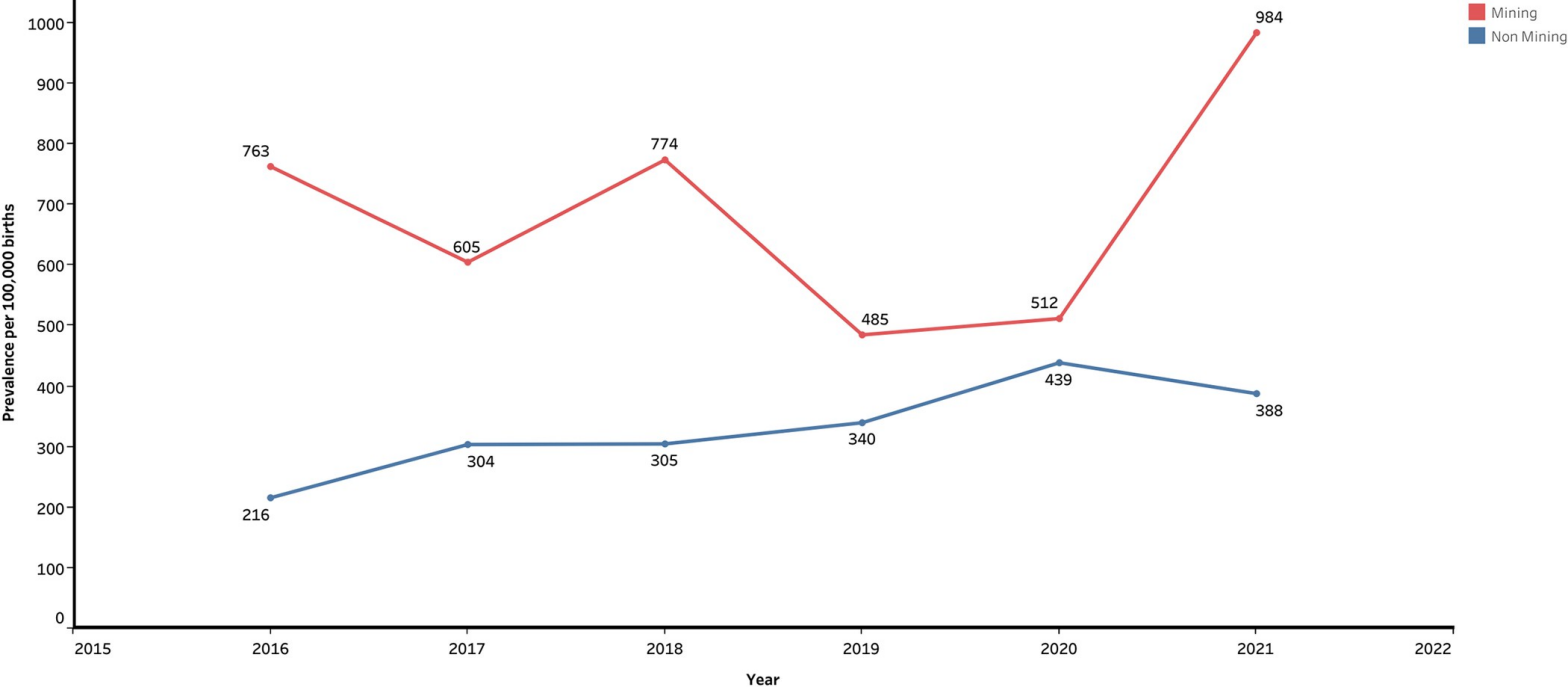
Line chart of the prevalence of BDs per 100,000 births in South Kivu. This chart displays the prevalence of BDs by mining status from 2016 to 2021.

A total of 261 cases of BDs were recorded on the study sites, accounting for an overall prevalence of 398.79 BDs per 100,000 births. Major BDs account for 166 (63.61%) cases. The prevalence of BDs in mining zones was found at 399 per 100,000 live births. The highest prevalence was found in Mwenga (1365 per 100,000 births) and in mining zones (666 per 100,000 births) (**Table 1**). The prevalence was significantly higher in Mubumbano compared to Walungu (Wilcoxon Signed Ranks Test=-3.69, *p*=*0*.*005*), in Mwenga compared to Walungu (-3.57, *p*<*0*.*001*), in Mumbumbano compared to Katana (-3.12, *p=0*.*042*), in Mwenga compared to Katana (-3.58, *p=0*.*007*), and in Mwenga compared to Mwana (-3.50, *p=0*.*009*). A higher prevalence was found in mining zones compared to non-mining zones (mean of prevalence 678 vs 326, t=4.13, *p<0*.*001*) though non-mining zones had a highest number of cases compared to mining zones (mean rank 1.69 vs 1.32, Friedman test=10.96, *p*<*0*.*001*). The distribution of BDs prevalences during the study period are summarized **Figs 1 and 2**.

**Table 1:** Prevalence of visible birth defects in South Kivu.

Health Zone/GRH	Number of new born with birth defects	Number of normal births	Prevalence per 100,000 births (95% CI)	Risk of occurrence (OR, 95%CI)
**No mining Zone**				
Walungu/FsKi	27	12424	217 (135-299)	0.49 (0.33-0.73)
Katana/Fomulac	24	8423	285 (171-398)	0.69 (0.45-1.04)
Ibanda/Panzi GRH	59	17259	341 (254-427)	0.79 (0.59-1.06)
Kadutu/HPGRB	55	12779	429 (316-542)	1.09 (0.82-1.48)
**Sub-group**	**165**	**50885**	**323 (274-372)**	**0.48 (0.37-0.62)**
**Mining zone**				
Mwana	22	5828	376 (219-534)	0.94 (0.61-1.45)
Mumbumbano	38	5898	640 (437-843)	**1.72 (1.22-2.43)**
Mwenga	36	2602	1365 (922-1807)	**3.89 (2.73-5.54)**
**Sub-group**	**96**	**14328**	**666 (533-798)**	**2.07 (1.61-2.67)**
**Overall**	**261**	**65213**	**399 (350-447)**	**-**

OR: Odds ratio, 95%CI: 95% confidence interval.

### Proportion and prevalence of CVA sub-groups

**Fig 3:**
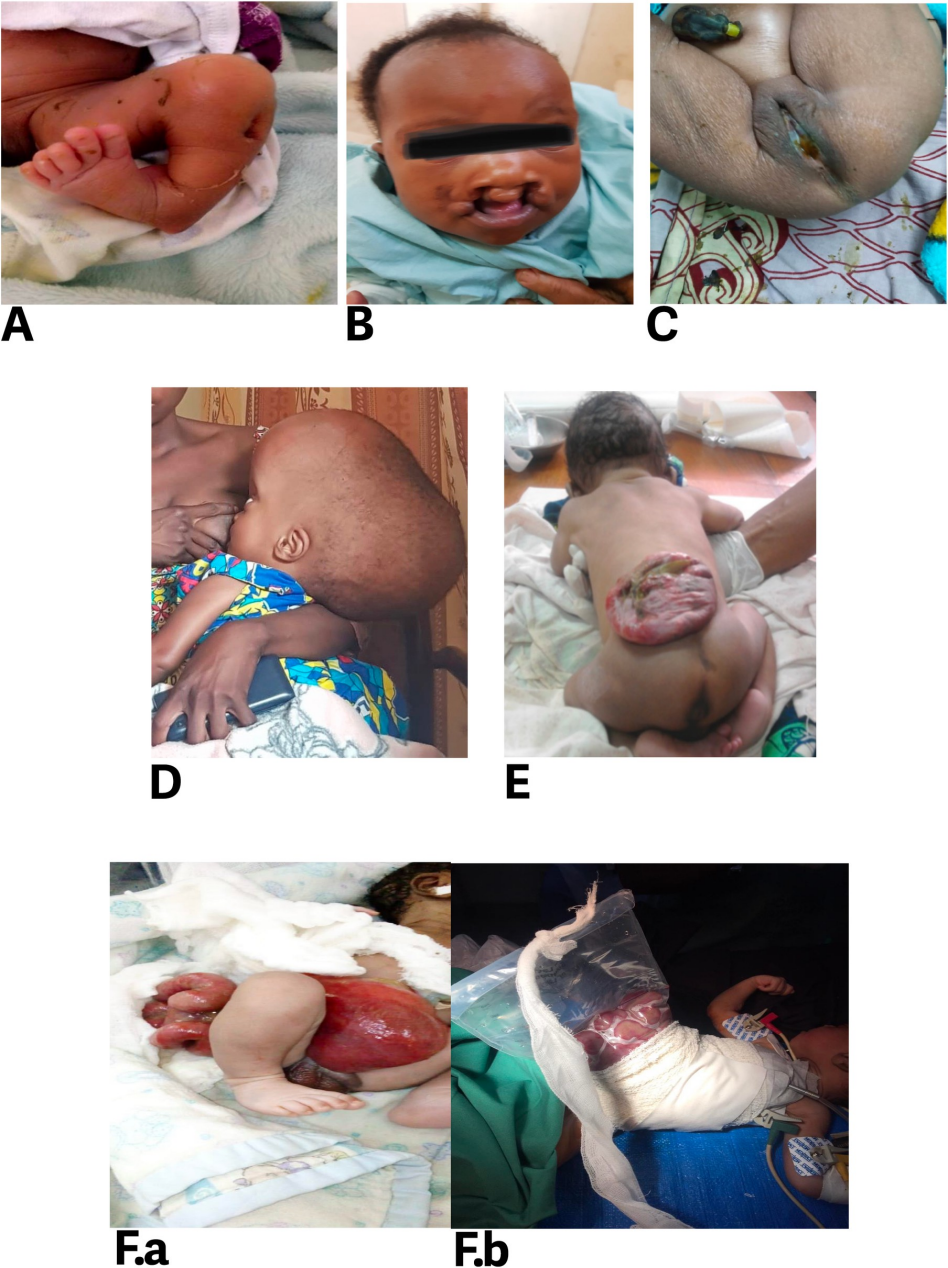
Birth defects diagnosed and managed in South Kivu. **(A)** A newborn with clubfoot diagnosed at birth and underwent successful treatment at the Provincial General Referral Hospital of Bukavu (HPGRB), located in Kadutu, in 2021. (**B**) An infant with bilateral cleft lip and palate underwent unsuccessful surgery at one week of age at a peripheral center and was subsequently referred for corrective surgery at HPGRB at the age of three months. (**C**) A baby with an anorectal malformation (imperforate anus) diagnosed at birth at Mwenga General Hospital and was referred for surgery at HPGRB on the third day of life. The surgery was successful. (**D**) An infant with a late diagnosis of hydrocephalus made at Fomulac Katana General Hospital. Subsequently, he was referred for surgery at HPGRB at the age of 8 months. (**E**) Newborn baby with spina bifida, diagnosed at birth and referred for surgery at the HPGRB the same day. The surgery was a success. (**F.a-b**) A neonate with laparoschisis diagnosed at birth in a peripheral center and experienced poor stabilization. Subsequently, he was referred for surgery at HPGRB 72 hours after birth, but unfortunately, he died 24 hours after the surgery.

BDs involving the musculoskeletal system (75, 28.74%) was the most common followed by those involving the central nervous system (CNS) (52, 19.92%), gastrointestinal (GI) system and the abdominal wall (47, 18.01%), polymalformation (45, 17.24%), face (35, 13.41%), and genitourinary system (7, 2.68%). Proportions of BDs involving the GI system and the abdominal wall were significantly different among mining and non-mining zones (*p*<0.001). The proportions of anorectal malformations (AMRs) (6.51% vs 3.07%) and agenesis of rectus abdominis (0.38% vs 0.00%) were higher in mining zones, while proportion of omphalocele (4.21% vs 1.53%) was higher in non-mining zones. BDs involving the face (97 vs 41 per 100,000 births), GI system and abdominal wall (173 vs 43 per 100,000 births), musculoskeletal system (166 vs 100 per 100,000 births), CNS (125 vs 67 per 100,000 births) and genitourinary system (28 vs 6 per 100,000 births) show a significant difference in prevalence between mining and non-mining zones (**S1 Fig**). Anorectal malformations, club foot, orofacial clefts, hydrocephalus and spina bifida were the most common BDs in mining zones accounting for 118, 97, 76, 62 and 62 per 100,000 births respectively. In non-mining zones, the most frequent BDs were polydactyly (55 per 100,000 births), orofacial clefts (41 per 100,000 births), club foot (29 per 100,000 births), dysraphisms such as hydrocephalus or spina bifida (25 per 100,000 births), respectively. Proportions and prevalence of BDs’ types are summarized in **Table 2** and illustrative cases of BDs diagnosed and operated on at the study sites are presented in **Fig 3**.

**Table 2:** Number and prevalence of birth defect types recorded in South Kivu.

System involved	Type	Number			Prevalence per 100,000 births	Frequency in %			*p*
		MZ	NMZ	Total		MZ	NMZ	Total	
Face		14	21	35	54	5.36	8.05	13.41	*0*.*67*
	Orofacial clefts	11	21	32	49	4.21	8.05	12.26	
	Anophthalmia	1	0	1	2	0.38	0	0.38	
	Mandibular agenesis	2	0	2	3	0.77	0	0.77	
Gastrointestinal system and abdominal wall		25	22	47	72	9.58	8.43	18.01	***<0*.*001***
	Anorectal malformation	17	8	25	38	6.51	3.07	9.58	
	Omphalocele	4	11	15	23	1.53	4.21	5.75	
	Laparoschisis	3	3	6	9	1.15	1.15	2.3	
	Agenesis of rectus abdominis	1	0	1	2	0.38	0	0.38	
Musculoskeletal system		24	51	75	115	9.2	19.54	28.74	*0*.*31*
	Polydactyly	7	28	35	54	2.68	10.73	13.41	
	Upper limbs agenesis	3	5	8	12	1.15	1.92	3.07	
	Club foot	14	15	29	44	5.36	5.75	11.11	
	Amniotic band syndrome (constriction ring syndrome)	0	2	2	3	0	0.77	0.77	
	Genu recurvatum	0	1	1	2	0	0.38	0.38	
Central Nervous System		18	34	52	80	6.9	13.03	19.92	*0*.*72*
	Hydrocephalus	9	13	22	33	3.45	4.98	8.43	
	Spina bifida	9	13	18	28	3.45	4.98	6.9	
	Anencephaly	0	8	8	12	0	3.07	3.07	
Genitourinary system		4	3	7	11	1.53	1.15	2.68	*0*.*26*
	Ambiguous genitalia	3	3	6	9.2	1.15	1.15	2.3	
	bladder exstrophy	1	0	1	1.53	0.38	0	0.38	
Polymalformation		11	34	45	69	4.21	13.03	17.24	*0*.*06*
	Hydrocephalus and Spina bifida	1	7	8	12	0.38	2.68	3.07	
	Club foot and Polydactyly	0	5	5	8	0	1.92	1.92	
	Spina bifida and Club foot	6	3	9	14	2.3	1.15	3.45	
	Orofacial clefts and Club foot	0	5	5	8	0	1.92	1.92	
	Orofacial clefts and polydactyly	1	2	3	5	0.38	0.77	1.15	
	Club foot and Ambiguous genitalia	1	1	2	3	0.38	0.38	0.77	
	Omphalocele and Orofacial clefts	0	2	2	3	0	0.77	0.77	
	Omphalocele, Orofacial clefts, and Club foot	0	1	1	2	0	0.38	0.38	
	Omphalocele and Spina bifida	1	3	4	6	0.38	1.15	1.53	
	Ambiguous genitalia and Anencephaly	0	1	1	2	0	0.38	0.38	
	Omphalocele and Ambiguous genitalia	0	1	1	2	0	0.38	0.38	
	Omphalocele and Upper limbs agenesis	1	1	2	3	0.38	0.38	0.77	
	Club foot and Upper limbs agenesis	0	1	1	2	0	0.38	0.38	
	Club foot and Laparoschisis	0	1	1	2	0	0.38	0.38	
Total		96	165	261	399	36.78	63.22	100	

MZ: Mining zones, NMZ: Non-mining zones (bold: Means statisticaly significant p-value).

**Fig 4:**
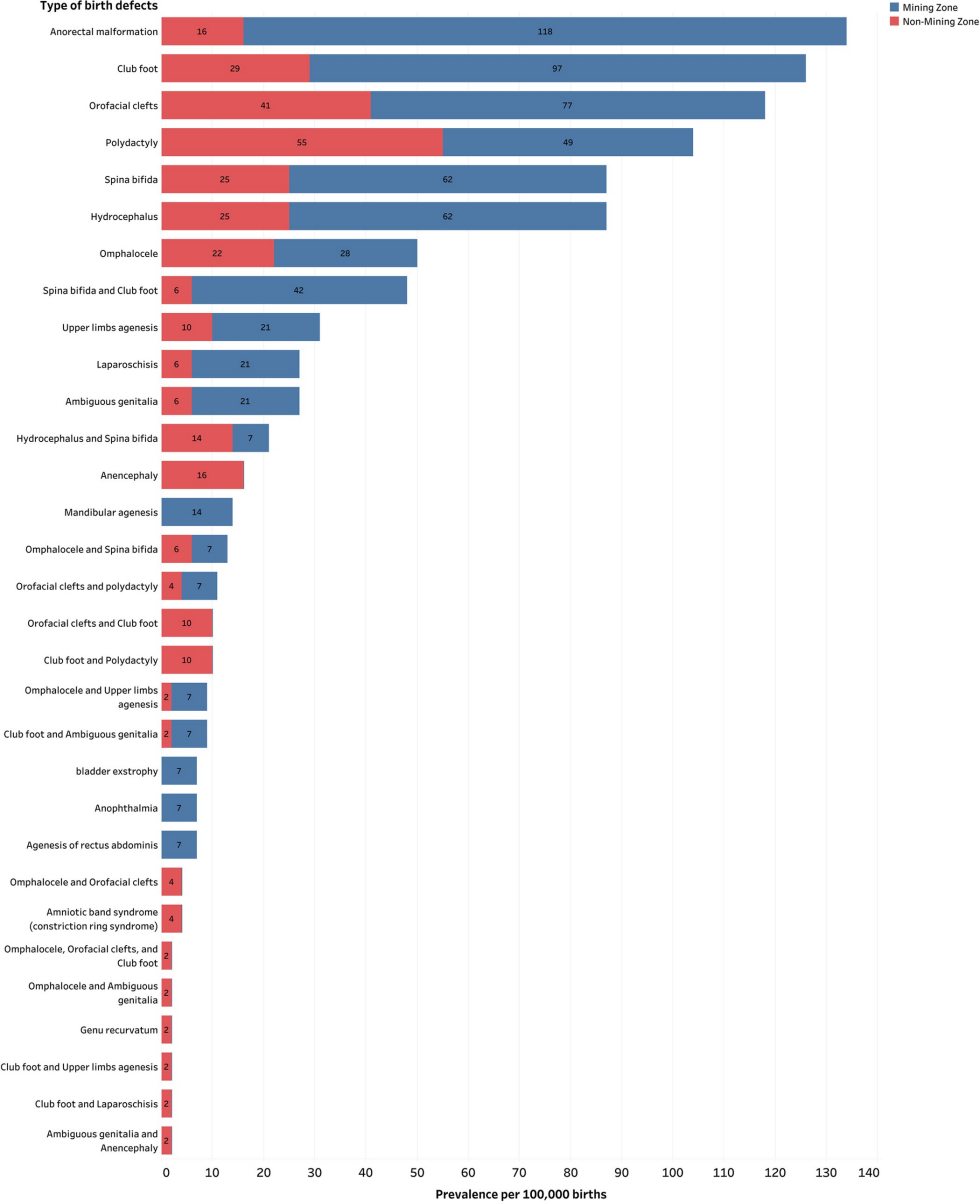
Total prevalence of reported type of BDs in relation to mining status.

**Fig 4** displays the prevalence total prevalence of BD’s types reported in study settings in relation to mining status. AMRs were the most prevalent in mining zones, with a total prevalence of 118 per 100,000 live births. In contrast, polydactyly (55 per 100,000 live births) was the most prevalent in non-mining zones.

### Risk of occurrence

**Fig 5:**
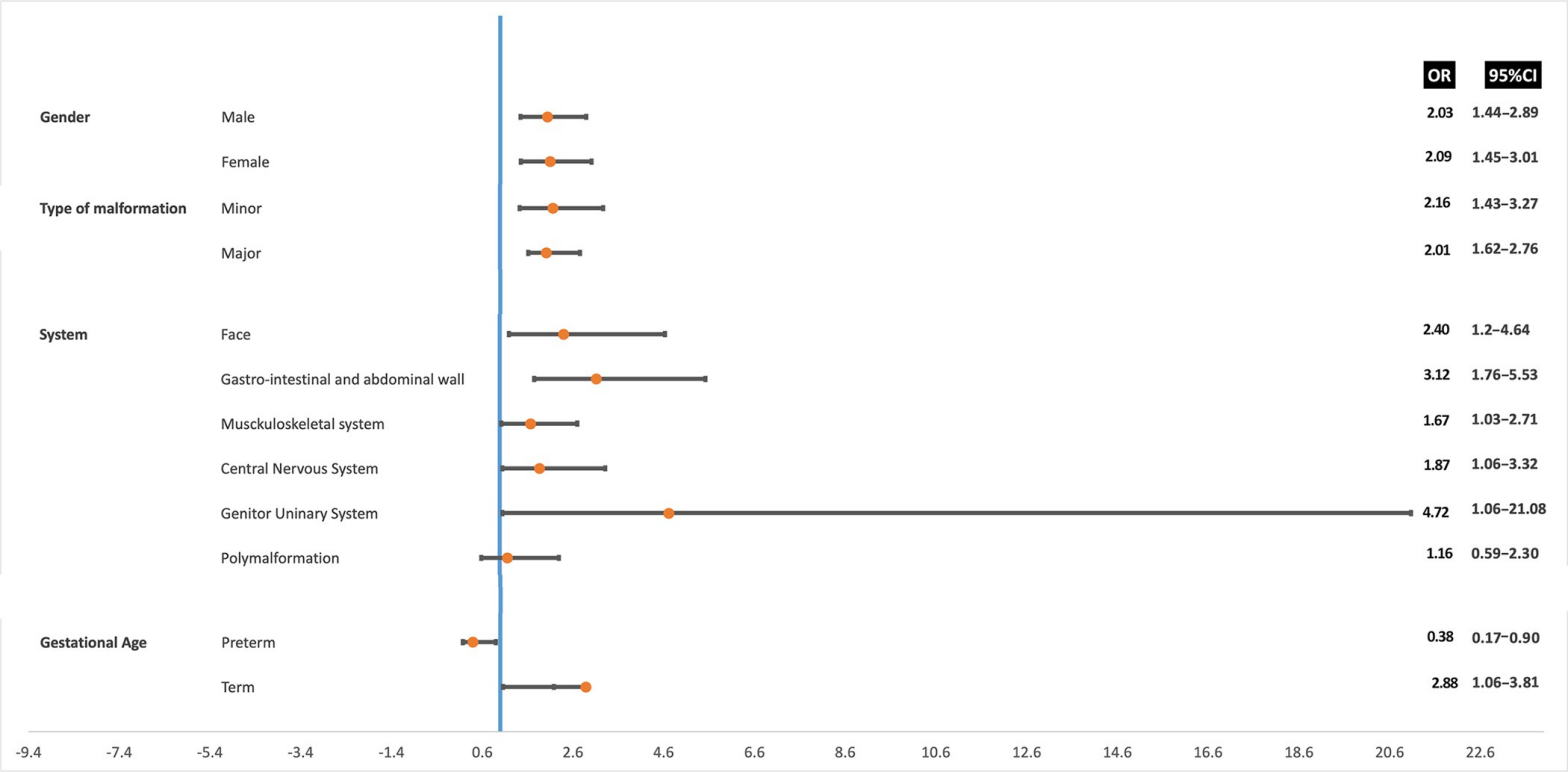
Forest plot displaying the risk of BDs occurrence by mining status, sex, type of malformation, system involved and the gestational age at birth. Odds ratio (OR) are represented by an orange ball with a related horizontal bar representing the 95% confidence interval. The vertical line (*red line*) represents the null value of the OR (1.00), and variables whose horizontal bar does not intersect the red line are significantly associated with the risk of BDs occurrence. The variables on the right are associated with an increase in risk while those on the left are associated with a significant decrease in risk. The reference category was “living non-mining zones”.

Univariate regression analysis was used to assess the association between BDs, types of BDs, sex, gestational age at birth, study site and mining status. We found that the risk of occurrence of BDs was two-folds in mining zones compared to non-mining zones (odds ratio[OR]=2.07, 95%CI[1.59-2.68]). In Mumbumbano and Mwenga the likelihood of BDs was significantly increased at two (OR=1.72, 95%CI[1.22-2.43]) and four (OR=3.89, 95%CI[2.73-5.54]) folds, while in Walungu it was decreased by almost half (OR=0.49, 95%CI[0.33-0.73]) (**Table 1**). **Fig 5** summarizes the risk of occurrence of BDs by sex, type of malformation, system involved and gestational age. Compared to neonates born to mothers in non-mining zones, we observed an elevated occurrence of BDs in those born to mothers in mining zones. Specifically, when considering sex, both females and males born to mothers in mining zones demonstrated heightened risks of BDs, with an OR of 2.09 (95%CI[1.45-3.01]) and 2.03 (95%CI[1.44-2.89]) respectively. Minor and major types of BDs were 2.01 (95%CI[1.43-3.27]) and 2.16 (95%CI[1.62-2.76]) times more likely to occur in mining. Depending on the system affected, BDs involving multiple systems (polymalformation) were the only ones not found to be significantly associated with mining (OR=1.16, 95%CI[0.59-2.30]). Additionally, term deliveries were associated with an increased risk of 3 (OR=2.88, 95%CI[1.06-2.18]).

## Discussion

This study comprehensively investigated the epidemiology of visible BDs in SK, an eastern mining-rich region of the DRC. It examined the prevalence and risk of occurrence in relation to mining activities. The findings offer detailed estimates of the distribution of BDs in both mining and non-mining areas in the region. Additionally, these estimates were compared to data from the Global Burden of Disease (GBD) study.

### Distribution of birth defects in South Kivu

Our findings revealed that nearly 4 out of every 1,000 live newborns in SK were born with BDs. This prevalence was lower than that reported in a similar study carried out in Lubumbashi, the mining capital of DRC, in 2009 of 6 per 1,000 births [15], and the national estimates reported by international organizations of 72 per 1,000 births in 2006 [26] and 27 to 65 per 1,000 births in 2019 [27]. The discrepancy between grounded-observations and modeled estimates can be partly attributed to the lack of a reliable data source notably a national birth defect registry in the country. This discrepancy could also be due to dissimilarity in the methodological approach, study period, design and setting.

The development and implementation of such a registry will not only improve the quality and quantity of research but enhance strategies for preventing and improving health outcomes in BDs. Nevertheless, our findings are consistent with the existing evidence reported in other Sub-Saharan African Countries [28–32]. Recent reports estimated that BDs affect 28 and 30 and in every 1,000 newborns in Europe [33], and the United States [34], respectively. The disparity between our findings and that of these highly performing countries might probably be due to the fact that we only focus on visible BDs. This limitation arises from the limited capacity, in this region, in diagnosing internal BDs which account for a significant proportion of BDs. For example, 3-4 out 10 BDs are congenital heart defects, however we didn’t take into account deliveries conducted at home. This observation reflects underestimation of the true epidemiological landscape and the burden of disease related to BDs in SK and the DRC. Such a phenomenon is heightened by the disparity in diagnosis capacity with limited capacity in rural and public hospitals.

We also pointed out the trend of the distribution of different types of BDs in SK. Our findings are consistent with previous estimates reporting a high prevalence of musculoskeletal BDs in the country [27,35,36]. However; they contrast with GBD data on BDs involving urogenital (11 per 1,000 births), neural (3 per 1,000 births), digestive (2 per 1,000 births) and orofacial systems(1 per 1,000 births) [27] (**S1 Table**). This comparison points the disparity between the modeled estimate and the on-the-ground observations. Our findings will possibly inform targeted interventions to reduce the burden of BDs in the DRC through prevention, epidemiological surveillance, and quality management. It is likely that in many areas of the DRC, BDs are still misdiagnosed or undiagnosed at birth, and may go undetected during the neonatal period. Moreover, these conditions are under-reported in the community due to the precariousness of the health information system and the lack of a national policy for monitoring and preventing them. Major BDs are increasingly reported to the disregard of minors, which are considered the neglected stepchild of newborns’ health by healthcare workers and the community. The hypothesis in support of this seems more likely considering a disparity in economic trends across provinces in the country. Nevertheless, it is not unusual for the pattern of BDs to vary from region to region [37], and for other reasons that are not yet fully understood. Previous studies have documented the worldwide variation between individual countries and regions [1,38–40]. Our findings highlight the need for larger epidemiological and population-based studies.

### Mining and birth defects

We observed a marked disparity according to mining status, with twice as many cases in mining zones compared with non-mining zones. The specific prevalence rates between regions revealed that cases of visible BDs were two to seven times higher in mining zones compared to non-mining zones. Our findings are consistent with a study conducted in Lubumbashi which reported that the cases of BDs were 1.6 times higher in facilities close to mining areas than for those around non-mining areas [16]. It is increasingly plausible that mining plays a role in the occurrence of BDs in the DRC. Women living in mining areas were twice more likely to deliver a newborn with a birth defect than those in non-mining areas in SK. This observation was further evidenced by subgroup analysis performed in our study and comparison between study sites. When we plotted the prevalence between sites, we found that they were higher in mining areas than in non-mining areas. The reported prevalence was two and three times higher in Mubumbano and five and six times higher in Mwenga than in Katana and Walungu respectively. In addition, living in Mubumbano and in Mwenga were risk factors for BDs while living in Katana was protective. In fact, these statistics are not at all surprising, since these two HZs are part of the areas where there are significant mining activities in SK. The contrasting pattern of BDs between non-mining and mining areas, and the association reported between GI system and the abdominal wall defects, suggest a relationship between the types of BDs and mining. However, while this association might indirectly suggest a relationship between gold mining exposure and the occurrence of AMRs, it would be presumptuous to support such a conclusion without substantial evidence. Therefore, it is crucial to investigate whether there is a correlation between human exposure to chemicals from gold mining—not limited to mercury—and the incidence of AMRs in the region. Furthermore, it’s imperative to emphasize that the observed association does not imply a causal relationship where mining directly causes birth defects. Further studies are needed to elucidate the true nature of the relationship between specific mining activities and specific types of BDs in the country.

In recent years, there has been an increasing recognition of chemical substances from mining such as mercury, lead, arsenic and more on the quality of reproduction, and development of BDs [41]. Intoxication from these chemicals in most cases, particularly in developing countries such the DRC, results from mining activities that are neither controlled nor regulated. The most obvious pathways are long-term inhalation of metals, as with mercury to separate gold with other concentrate, and poisoning of the environment including air and water pollution, water by mineral processing wastes and crushed ore [9,42]. Indeed, the soils and sediments of water points near artisanal farm sites and places where the treatment of gold with mercury is carried out are polluted from artisanal and small-scale gold mining [43–45]. This process, widespread in SK [46], in which mercury is mixed with gold-containing materials, forming a mercury-gold amalgam which is then heated, vaporizing the mercury to obtain the gold [47], is highly hazardous and can lead to significant mercury exposure and health risks, including birth defects [48]. A recent meta-analysis suggested a significant association between maternal mercury exposure, including drinking mercy-containing water, and specific congenital heart defects [49].

Another pathway is the consumption of food polluted by heavy metals. The kapolowe river serves mining areas with high concentrations of heavy metals and is also the major source of fresh fish called “kapolowe” in Lubumbashi [50]. A significant association was observed between regular consumption of Kapolowe fish in the first trimester of pregnancy and the appearance of orofacial cleft. The hypothesis underlying this observation posits that the fishes contain a high concentration of heavy metals and therefore contributing to the occurrence of orofacial clefts [51]. As a result of long-term exposure and slow elimination in the human body, heavy metals might cross the placental-maternal barrier and impact fetus health [52]. Thus, from various mechanisms such as teratogenesis or chromosomal damage they increase the likelihood of miscarriage, stillbirth, fetal growth retardation or newborn with BDs [53]. Accumulation of heavy metals to toxic level can have harmful effects on the male reproductive system resulting in poor sperm quality and decreased sperm function [54,55]. A case-control study suggests a relationship between urinary arsenic and cadmium concentrations and accelerated and severe alteration of spermatozoa, cells that carry genetic materials [56]. Evidence on mining fathers’ exposure emphasizes the contribution of epigenetic alterations in spermatozoa could impact on postfertilization gene transcriptional regulation in offspring tissues [57–61].

Finally, exposure to ionizing radiation can interfere with embryonic development and increase the risk of BDs. The geo-mineralogical environment of SK is rich in coltan [62], known as blood ore, a naturally radioactive mineral whose radiation levels are significantly higher than those observed in the natural environment. Artisans working on coltan grinding and sieving are potentially exposed to high levels of occupational exposure to naturally occurring radioactive materials “NORM”, due to working conditions and the radioactivity of coltan [63]. Occupational radiation exposures are also detected in the mining of other minerals. Along the Ulindi River in SK, high environmental radioactivity has been detected in artisanal and small-scale gold mining [64].

Despite the importance of this study to provide an overview of BDs in South Kivu, it may explicitly lead to bias. We collected retrospective data from hospital records in which the records were incomplete or information was partly missing from the record. Also, only deliveries occurring in referral hospitals were recorded, suggesting a recruitment bias. Deliveries taking place in lower-level health facilities, such health centers, or private hospitals were not recruited. Moreover, this study is subject to ecological fallacy and misinterpretation biases. Individual characteristics, behaviors, and BDs risk factors may not only vary between people living in mining and non-mining zones, but also within people living in the same zone. It is not excluded that relying solely on clinical diagnoses may introduce variability in the identification and categorization of congenital malformations. Standardization across hospitals can vary, impacting the precision of prevalence rates. Due to the retrospective design of the study, we failed to account for confounding variables or extraneous factors such as time living in mining zones, working and/or living in mining zones, environmental level of metal poisoning, supplementation in iron and folic acid, prenatal care and more. A substantial amount of data pertaining to these variables was lacking in the medical records, leading to their exclusion from the statistical analyses. Consequently, the reported results may not portray the accurate prevalence and risk of occurrence of BDs in SK.

Despite this limitation, this study provides a range of valuable information on the prevalence of BDs and its association with mining in SK that will allow stakeholders to strengthen the health system through research, advocacy, and practice. First, we reviewed a large population of 65,474 deliveries. The study findings can be generalized to the SK population. Given the study’s duration of over 6 years, it allowed to establish a temporal pattern of the outcome and a temporal relation between the outcome and the factors. Second, due to the contextual similarities across the DRC, the results of this study would form the basis for the development of a national policy for the prevention and management of BDs. Indirectly, it highlights areas that should be developed and strengthened, especially diagnosis, genetics and evidence-based prevention. Likewise, this study also suggests the need for a policy aimed at regulating mining activities to mitigate their detrimental impact on health. The Congolese health system should leverage the existence of high-level and international health policies such the World Health Assembly Resolution 79.16 on food fortification with acid folic to prevent BDs [65,66]. Such initiatives are mandatory to further and support the achievement of the sustainable development goals (SDGs) agenda in the country, especially the SDG target 3.2 “*End preventable deaths of newborns and children under 5 years of age*”.

BDs pose challenges related to limited healthcare access, societal stigma, and the need for robust social support. Addressing these issues requires comprehensive strategies, including improved healthcare infrastructure, awareness campaigns, and community engagement initiatives. Third, public health policies in the region should address not only clinical aspects of neonate and maternal health but also environmental risks associated with mining. The regional disparities in BD prevalence necessitate targeted interventions, particularly in high-prevalence areas. Strategies focusing on maternal health, early detection, and specialized care can contribute to mitigating the impact of BDs. Robust environmental monitoring, risk assessments, and stringent regulations are crucial. Collaboration among health authorities, environmental agencies, and mining stakeholders is indispensable. Cross-disciplinary and holistic health system investments will improve BD research, diagnosis, and management. This study serves as a platform for advocacy to enhance environmental health standards in mining zones, promoting awareness, community engagement, and policy advocacy. These efforts foster a comprehensive understanding of environmental risks, encouraging proactive measures to protect the health of the population, especially pregnant women and newborns. Additionally, ongoing monitoring and further research are essential to refine policies and enhance understanding. Future research should capitalize on the insights gained from the limitations of this study to thoroughly investigate the association between mining and BDs. Additionally, the measurement of association should be adjusted for the aforementioned confounding factors.

## Conclusion

The epidemiology of visible BDs in this study reveals a low prevalence compared to national estimates and international reported data. Major BDs and musculoskeletal BDs were the most common types, suggesting that non-severe and inconspicuous types are underreported. BDs were twice as likely to occur in mining areas compared to non-mining areas in this study. The mismatched profile of BD types in these two areas reinforces the impact of mining on BDs. It also hints at the correlation of a particular mining activity with specific BD types. However, further research with robust analysis and study design, integrating contaminants, are needed to validate our findings.

This study calls for action towards more robust and advanced study designs in response to the urgent need to increase the quality and quantity of BD research in South Kivu and the DRC. Local research institutions such as the Center for Tropical Diseases and Global Health (CTDGH) at the UCB should leverage their relationships with sister institutions in Europe and America involved in BD research and prevention, such as KU Leuven, Global Alliance for Prevention of Spina Bifida - F (GAPSBi-F), and SUNY Downstate Health Science University, to improve expertise in research and draw industry support to the DRC. In addition, our findings provide insight into priorities for future research collaborations. We hypothesize the environmental impact on newborn health and identify a need for genetic and epigenetic research and the development and implementation of a BD registry. The CTDGH should leverage this opportunity to champion such initiatives. Thus, interventions to improve BD care should be based on reliable evidence.

## Supporting information

S1 Table Supplemental table Cikomola BDs. This table presents birth defects data for the Democratic Republic of Congo from the Global Burden of Disease Study 2019.
(DOCX)

S1 Fig This figure displays the prevalence of visual birth defects per category in relation to mining status. (TIFF)

S1 File Cikomola Normal births BDs dataset. This table provides detailed monthly birth statistics and the total number of birth defects for each study site.
(XLSX)

S2 File Cikomola BDs dataset. This file contains all the deidentified raw data for visual birth defects.
(XLSX)

## Abbreviations

95%CI: 95% Confidence interval
AMRs: Anorectal malformations
BDs: Birth defects
CNS: Central nervous system
CTDGH: Center for Tropical Disease and Global Health
DALYs: Disability adjusted-life style years
DRC: Democratic Republic of Congo
GAPSBi-F: Global Alliance for Prevention of Spina Bifida-F
GBD: Global Burden of Disease
GI: Gastrointestinal
HPGRB: Hôpital Provincial Général de Référence de Bukavu
HZ: Health zone
IRB: Institutional Review Board
MZ: Mining Zone
NMZ: No-mining zone
OR: Odds Ratio
p: p-value
SDG: Sustainable development goals
SK: South Kivu
U.C.B: Catholic University of Bukavu
