# Supplementary material for: Prevalence and risk of occurrence of visible birth defects in mining areas in South Kivu: A hospital-based cross-sectional study
Source: PLoS One. 2024 Oct 7;19(10):e0309004. doi: 10.1371/journal.pone.0309004 (PMC11457993; doi:10.1371/journal.pone.0309004)
Supplement: S1 Table — This table presents birth defects data for the Democratic Republic of Congo from the Global Burden of Disease Study 2019. (DOCX) [file pone.0309004.s001.docx]

**Supplemental Table. Birth Defects Data for the Democratic Republic of Congo from the Global Burden of Disease Study 2019**

| **Metric** | **Gender** | **Age** | **Birth defects** | **Estimates** | **year** | **val** | **upper** | **lower** |
| --- | --- | --- | --- | --- | --- | --- | --- | --- |
| Deaths | Both | All ages | Congenital birth defects | Number | 2019 | 11692.3800 | 19445.4547 | 7107.3189 |
| Deaths | Both | All ages | Congenital birth defects | Percent | 2019 | 0.0200 | 0.0338 | 0.0132 |
| Deaths | Both | All ages | Congenital birth defects | Rate | 2019 | 13.3300 | 22.1802 | 8.1069 |
| Deaths | Both | All ages | Neural tube defects | Number | 2019 | 3288.4500 | 5902.3629 | 1658.5407 |
| Deaths | Both | All ages | Neural tube defects | Percent | 2019 | 0.0058 | 0.0102 | 0.0030 |
| Deaths | Both | All ages | Neural tube defects | Rate | 2019 | 3.7509 | 6.7324 | 1.8918 |
| Deaths | Both | All ages | Orofacial clefts | Number | 2019 | 81.8193 | 241.7151 | 24.1405 |
| Deaths | Both | All ages | Orofacial clefts | Percent | 2019 | 0.0001 | 0.0004 | 0.0000 |
| Deaths | Both | All ages | Orofacial clefts | Rate | 2019 | 0.0933 | 0.2757 | 0.0275 |
| Deaths | Both | All ages | Congenital musculoskeletal and limb anomalies | Number | 2019 | 310.0058 | 550.6122 | 158.3213 |
| Deaths | Both | All ages | Congenital musculoskeletal and limb anomalies | Percent | 2019 | 0.0005 | 0.0009 | 0.0003 |
| Deaths | Both | All ages | Congenital musculoskeletal and limb anomalies | Rate | 2019 | 0.3536 | 0.6280 | 0.1806 |
| Deaths | Both | All ages | Urogenital congenital anomalies | Number | 2019 | 214.2381 | 384.7990 | 109.8790 |
| Deaths | Both | All ages | Urogenital congenital anomalies | Percent | 2019 | 0.0004 | 0.0007 | 0.0002 |
| Deaths | Both | All ages | Urogenital congenital anomalies | Rate | 2019 | 0.2444 | 0.4389 | 0.1253 |
| Deaths | Both | All ages | Digestive congenital anomalies | Number | 2019 | 1259.1143 | 2524.5846 | 570.8059 |
| Deaths | Both | All ages | Digestive congenital anomalies | Percent | 2019 | 0.0022 | 0.0045 | 0.0010 |
| Deaths | Both | All ages | Digestive congenital anomalies | Rate | 2019 | 1.4362 | 2.8796 | 0.6511 |
| Deaths | Both | All ages | Other congenital birth defects | Number | 2019 | 2936.2433 | 6148.2568 | 1468.9679 |
| Deaths | Both | All ages | Other congenital birth defects | Percent | 2019 | 0.0052 | 0.0110 | 0.0027 |
| Deaths | Both | All ages | Other congenital birth defects | Rate | 2019 | 3.3492 | 7.0129 | 1.6756 |
| DALYs | Both | All ages | Congenital birth defects | Number | 2019 | 1117547.130 | 1793491.889 | 720109.958 |
| DALYs | Both | All ages | Congenital birth defects | Percent | 2019 | 0.0298 | 0.0469 | 0.0198 |
| DALYs | Both | All ages | Congenital birth defects | Rate | 2019 | 1274.7137 | 2045.7201 | 821.3828 |
| DALYs | Both | All ages | Neural tube defects | Number | 2019 | 300671.5135 | 530610.4187 | 155773.3128 |
| DALYs | Both | All ages | Neural tube defects | Percent | 2019 | 0.0080 | 0.0137 | 0.0043 |
| DALYs | Both | All ages | Neural tube defects | Rate | 2019 | 342.9565 | 605.2330 | 177.6805 |
| DALYs | Both | All ages | Orofacial clefts | Number | 2019 | 9756.3819 | 23485.5833 | 4432.9666 |
| DALYs | Both | All ages | Orofacial clefts | Percent | 2019 | 0.0003 | 0.0006 | 0.0001 |
| DALYs | Both | All ages | Orofacial clefts | Rate | 2019 | 11.1285 | 26.7885 | 5.0564 |
| DALYs | Both | All ages | Congenital musculoskeletal and limb anomalies | Number | 2019 | 70224.7153 | 97562.1534 | 47685.7479 |
| DALYs | Both | All ages | Congenital musculoskeletal and limb anomalies | Percent | 2019 | 0.0019 | 0.0026 | 0.0013 |
| DALYs | Both | All ages | Congenital musculoskeletal and limb anomalies | Rate | 2019 | 80.1008 | 111.2828 | 54.3920 |
| DALYs | Both | All ages | Urogenital congenital anomalies | Number | 2019 | 22667.6031 | 38643.7242 | 12847.5018 |
| DALYs | Both | All ages | Urogenital congenital anomalies | Percent | 2019 | 0.0006 | 0.0010 | 0.0004 |
| DALYs | Both | All ages | Urogenital congenital anomalies | Rate | 2019 | 25.8555 | 44.0784 | 14.6543 |
| DALYs | Both | All ages | Digestive congenital anomalies | Number | 2019 | 111566.4970 | 222984.1690 | 50646.6665 |
| DALYs | Both | All ages | Digestive congenital anomalies | Percent | 2019 | 0.0030 | 0.0061 | 0.0014 |
| DALYs | Both | All ages | Digestive congenital anomalies | Rate | 2019 | 127.2567 | 254.3436 | 57.7694 |
| DALYs | Both | All ages | Other congenital birth defects | Number | 2019 | 280116.9529 | 557107.2985 | 151592.3803 |
| DALYs | Both | All ages | Other congenital birth defects | Percent | 2019 | 0.0075 | 0.0154 | 0.0040 |
| DALYs | Both | All ages | Other congenital birth defects | Rate | 2019 | 319.5113 | 635.4562 | 172.9116 |
| Prevalence | Both | <5 years | Digestive congenital anomalies | Number | 2019 | 9227.0374 | 12565.9254 | 6454.5624 |
| Prevalence | Both | <5 years | Digestive congenital anomalies | Percent | 2019 | 0.0007 | 0.0010 | 0.0005 |
| Prevalence | Both | <5 years | Digestive congenital anomalies | Rate | 2019 | 67.6992 | 92.1968 | 47.3574 |
| Prevalence | Both | <28 days | Urogenital congenital anomalies | Number | 2019 | 2040.1630 | 3010.0683 | 1342.9770 |
| Prevalence | Both | <28 days | Urogenital congenital anomalies | Percent | 2019 | 0.0107 | 0.0157 | 0.0070 |
| Prevalence | Both | <28 days | Urogenital congenital anomalies | Rate | 2019 | 927.1743 | 1367.9584 | 610.3305 |
| Prevalence | Both | <5 years | Congenital musculoskeletal and limb anomalies | Number | 2019 | 124063.1450 | 174432.7305 | 81014.4654 |
| Prevalence | Both | <5 years | Congenital musculoskeletal and limb anomalies | Percent | 2019 | 0.0095 | 0.0134 | 0.0062 |
| Prevalence | Both | <5 years | Congenital musculoskeletal and limb anomalies | Rate | 2019 | 910.2569 | 1279.8208 | 594.4068 |
| Prevalence | Both | <5 years | Urogenital congenital anomalies | Number | 2019 | 58331.8797 | 78709.8077 | 43060.0979 |
| Prevalence | Both | <5 years | Urogenital congenital anomalies | Percent | 2019 | 0.0045 | 0.0061 | 0.0033 |
| Prevalence | Both | <5 years | Urogenital congenital anomalies | Rate | 2019 | 427.9836 | 577.4974 | 315.9339 |
| Prevalence | Both | <28 days | Congenital musculoskeletal and limb anomalies | Number | 2019 | 4644.2768 | 6712.0450 | 2846.9294 |
| Prevalence | Both | <28 days | Congenital musculoskeletal and limb anomalies | Percent | 2019 | 0.0242 | 0.0349 | 0.0150 |
| Prevalence | Both | <28 days | Congenital musculoskeletal and limb anomalies | Rate | 2019 | 2110.6423 | 3050.3621 | 1293.8181 |
| Prevalence | Both | <28 days | Digestive congenital anomalies | Number | 2019 | 354.2366 | 472.8006 | 255.2485 |
| Prevalence | Both | <28 days | Digestive congenital anomalies | Percent | 2019 | 0.0018 | 0.0025 | 0.0013 |
| Prevalence | Both | <28 days | Digestive congenital anomalies | Rate | 2019 | 160.9867 | 214.8694 | 116.0005 |
| Prevalence | Both | <5 years | Other congenital birth defects | Number | 2019 | 22405.2749 | 39715.2373 | 11742.4411 |
| Prevalence | Both | <5 years | Other congenital birth defects | Percent | 2019 | 0.0017 | 0.0031 | 0.0009 |
| Prevalence | Both | <5 years | Other congenital birth defects | Rate | 2019 | 164.3885 | 291.3925 | 86.1548 |
| Prevalence | Both | <28 days | Other congenital birth defects | Number | 2019 | 362.0240 | 641.5744 | 189.7742 |
| Prevalence | Both | <28 days | Other congenital birth defects | Percent | 2019 | 0.0019 | 0.0034 | 0.0010 |
| Prevalence | Both | <28 days | Other congenital birth defects | Rate | 2019 | 164.5257 | 291.5705 | 86.2449 |
| Prevalence | Both | <28 days | Congenital birth defects | Number | 2019 | 14220.9376 | 16686.2020 | 11969.5501 |
| Prevalence | Both | <28 days | Congenital birth defects | Percent | 2019 | 0.0743 | 0.0872 | 0.0623 |
| Prevalence | Both | <28 days | Congenital birth defects | Rate | 2019 | 6462.8603 | 7583.2267 | 5439.6927 |
| Prevalence | Both | <5 years | Neural tube defects | Number | 2019 | 13146.5462 | 16807.4033 | 10109.7185 |
| Prevalence | Both | <5 years | Neural tube defects | Percent | 2019 | 0.0010 | 0.0013 | 0.0008 |
| Prevalence | Both | <5 years | Neural tube defects | Rate | 2019 | 96.4568 | 123.3167 | 74.1755 |
| Prevalence | Both | <5 years | Orofacial clefts | Number | 2019 | 9835.2932 | 12675.9944 | 7549.4511 |
| Prevalence | Both | <5 years | Orofacial clefts | Percent | 2019 | 0.0008 | 0.0010 | 0.0006 |
| Prevalence | Both | <5 years | Orofacial clefts | Rate | 2019 | 72.1620 | 93.0043 | 55.3907 |
| Prevalence | Both | <5 years | Congenital birth defects | Number | 2019 | 362051.6618 | 426824.8621 | 302398.8167 |
| Prevalence | Both | <5 years | Congenital birth defects | Percent | 2019 | 0.0279 | 0.0329 | 0.0232 |
| Prevalence | Both | <5 years | Congenital birth defects | Rate | 2019 | 2656.3894 | 3131.6333 | 2218.7137 |
| Prevalence | Both | <28 days | Neural tube defects | Number | 2019 | 467.7865 | 603.2512 | 353.8381 |
| Prevalence | Both | <28 days | Neural tube defects | Percent | 2019 | 0.0024 | 0.0031 | 0.0018 |
| Prevalence | Both | <28 days | Neural tube defects | Rate | 2019 | 212.5907 | 274.1541 | 160.8056 |
| Prevalence | Both | <28 days | Orofacial clefts | Number | 2019 | 246.3302 | 373.9603 | 147.6504 |
| Prevalence | Both | <28 days | Orofacial clefts | Percent | 2019 | 0.0013 | 0.0020 | 0.0008 |
| Prevalence | Both | <28 days | Orofacial clefts | Rate | 2019 | 111.9475 | 169.9503 | 67.1013 |
| Incidence | Both | <28 days | Orofacial clefts | Number | 2019 | 0.0000 | 0.0000 | 0.0000 |
| Incidence | Both | <28 days | Orofacial clefts | Percent | 2019 | 0.0000 | 0.0000 | 0.0000 |
| Incidence | Both | <28 days | Orofacial clefts | Rate | 2019 | 0.0000 | 0.0000 | 0.0000 |
| Incidence | Both | <28 days | Congenital birth defects | Number | 2019 | 0.0000 | 0.0000 | 0.0000 |
| Incidence | Both | <28 days | Congenital birth defects | Percent | 2019 | 0.0000 | 0.0000 | 0.0000 |
| Incidence | Both | <28 days | Congenital birth defects | Rate | 2019 | 0.0000 | 0.0000 | 0.0000 |
| Incidence | Both | <5 years | Urogenital congenital anomalies | Number | 2019 | 27932.0473 | 41174.0444 | 18348.5154 |
| Incidence | Both | <5 years | Urogenital congenital anomalies | Percent | 2019 | 0.0002 | 0.0003 | 0.0001 |
| Incidence | Both | <5 years | Urogenital congenital anomalies | Rate | 2019 | 204.9387 | 302.0958 | 134.6239 |
| Incidence | Both | <28 days | Urogenital congenital anomalies | Number | 2019 | 0.0000 | 0.0000 | 0.0000 |
| Incidence | Both | <28 days | Urogenital congenital anomalies | Percent | 2019 | 0.0000 | 0.0000 | 0.0000 |
| Incidence | Both | <28 days | Urogenital congenital anomalies | Rate | 2019 | 0.0000 | 0.0000 | 0.0000 |
| Incidence | Both | <5 years | Neural tube defects | Number | 2019 | 7691.9986 | 9787.8870 | 5936.6651 |
| Incidence | Both | <5 years | Neural tube defects | Percent | 2019 | 0.0001 | 0.0001 | 0.0000 |
| Incidence | Both | <5 years | Neural tube defects | Rate | 2019 | 56.4365 | 71.8142 | 43.5576 |
| Incidence | Both | <5 years | Orofacial clefts | Number | 2019 | 3319.4522 | 5031.8657 | 1992.5043 |
| Incidence | Both | <5 years | Orofacial clefts | Percent | 2019 | 0.0000 | 0.0000 | 0.0000 |
| Incidence | Both | <5 years | Orofacial clefts | Rate | 2019 | 24.3550 | 36.9190 | 14.6191 |
| Incidence | Both | <5 years | Digestive congenital anomalies | Number | 2019 | 5162.0840 | 6791.3822 | 3777.2855 |
| Incidence | Both | <5 years | Digestive congenital anomalies | Percent | 2019 | 0.0000 | 0.0001 | 0.0000 |
| Incidence | Both | <5 years | Digestive congenital anomalies | Rate | 2019 | 37.8744 | 49.8287 | 27.7141 |
| Incidence | Both | <5 years | Congenital birth defects | Number | 2019 | 214218.8015 | 256062.6774 | 178382.5089 |
| Incidence | Both | <5 years | Congenital birth defects | Percent | 2019 | 0.0017 | 0.0021 | 0.0014 |
| Incidence | Both | <5 years | Congenital birth defects | Rate | 2019 | 1571.7330 | 1878.7434 | 1308.8005 |
| Incidence | Both | <5 years | Congenital musculoskeletal and limb anomalies | Number | 2019 | 63109.0661 | 91243.8885 | 38742.2090 |
| Incidence | Both | <5 years | Congenital musculoskeletal and limb anomalies | Percent | 2019 | 0.0005 | 0.0007 | 0.0003 |
| Incidence | Both | <5 years | Congenital musculoskeletal and limb anomalies | Rate | 2019 | 463.0341 | 669.4605 | 284.2533 |
