# Supplementary figures and images for: Prevalence and risk of occurrence of visible birth defects in mining areas in South Kivu: A hospital-based cross-sectional study
Source: PLoS One. 2024 Oct 7;19(10):e0309004. doi: 10.1371/journal.pone.0309004 (PMC11457993; doi:10.1371/journal.pone.0309004)

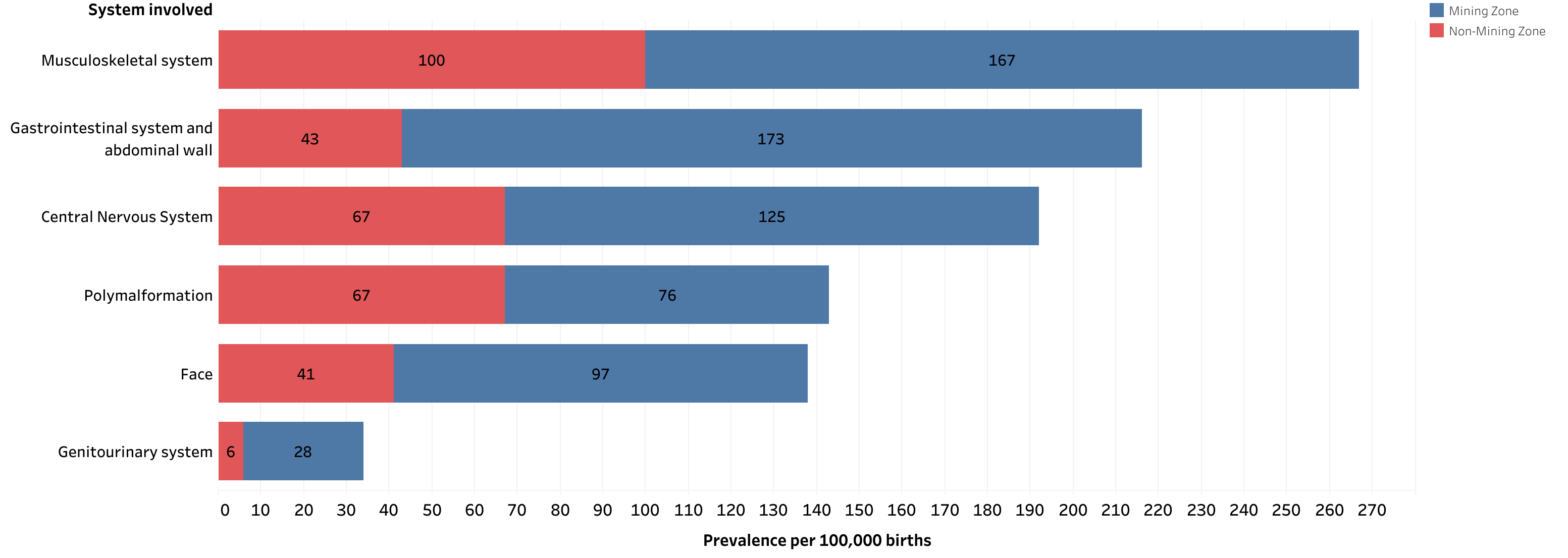

Supplement: S1 Fig — (TIFF) [file pone.0309004.s002.tiff]
